# The establishment of the population pharmacokinetic model needs to further address clinical needs while ensuring the standardization of data collection

**DOI:** 10.1128/aac.00678-25

**Published:** 2025-07-21

**Authors:** Lihui Wang, Yue Chen, Chunhui Xu, Mi Zhou, Runjie Li, Bin Lin, Yuetian Yu

**Affiliations:** 1 Department of Critical Care Medicine, Renji Hospital, Shanghai Jiao Tong University, School of Medicine 659335, Shanghai, China; 2 Department of Pharmacy, China-Japan Friendship Hospital 36635, Beijing, China; 3 State Key Laboratory of Experimental Hematology, National Clinical Research Center for Blood Diseases, Haihe Laboratory of Cell Ecosystem, Institute of Hematology and Blood Diseases Hospital, Chinese Academy of Medical Sciences & Peking Union Medical College 70585, Tianjin, China; 4 Department of Pharmacy, Children's Hospital of Soochow University 71532, Suzhou, China; 5 Key Laboratory of Intelligent Pharmacy and Individualized Therapy of Huzhou, Huzhou, China; 6 Department of Pharmacy, Changxing People's Hospital, Changxing Branch, Second Affiliated Hospital Zhejiang University School of Medicine 89681, Huzhou, China; Providence Portland Medical Center, Portland, Oregon, USA

**Keywords:** polymyxin B, population pharmacokinetics, nebulized, pneumonia

## LETTER

We appreciate the efforts of Li et al. in establishing a population pharmacokinetic model for nebulized Polymyxin B in patients with pneumonia caused by multidrug-resistant Gram-negative bacteria (1). This study provides evidence for the rational adjustment of related antibiotic dosages in clinical practice, ensuring pharmacokinetics/pharmacodynamics targets are achieved, and ultimately improving patient outcomes. However, there are still some methodological details in this study that require further discussion to enhance the precision of the model.

Accurately detecting the concentration of nebulized antibiotics in the epithelial lining fluid (ELF) has always been a challenging task, as it is difficult to standardize the dilution of bronchoalveolar lavage fluid (BALF) during bronchoalveolar lavage (BAL). Although urea can serve as an endogenous parameter to correct the dilution of BALF and measure the concentration of many soluble substances in the ELF (2), its results still differ significantly from those obtained using the gold standard microdialysis catheters. Jayesh’s study demonstrated that, in mechanically ventilated healthy sheep, the concentration of nebulized tobramycin in the ELF, after correction with urea, was more than 100 times higher than that measured by the microdialysis catheters (3). The primary reason for this discrepancy is the unavoidable contamination from nebulized antibiotic particles adhering to the bronchial walls during BAL. Typically, the outer diameter of the bronchoscope’s distal end is 4 mm, allowing access to the fourth-level bronchi (e.g., the A subsegment of the apical segment of the right upper lobe), while alveoli are located at the 23rd level of bronchi. Therefore, antibiotic particles in the bronchi from levels 5 to 22 result in a significantly higher concentration of nebulized tobramycin in the ELF, as corrected by urea, compared to the microdialysis catheters. Although many research methods suggest discarding the first bronchoalveolar lavage fluid, contamination of the results cannot be completely avoided. In this study, six BALF samples need to be collected every 2 hours after nebulization. It can be anticipated that the first two samples will be most affected by residual antibiotic concentrations on the bronchial walls, with subsequent contamination gradually decreasing. Consequently, the samples collected for concentration detection are not at the same baseline level, which affects the precision of model construction.

In addition, we must pay attention to the methodological consistency of using antibiotic nebulization during mechanical ventilation. In Li’s study, patients used either a vibration mesh nebulizer or a jet nebulizer (1); however, the efficiency of these two nebulizers differs significantly, impacting the effective concentration of nebulized antibiotics. If a jet nebulizer is used, approximately 51.5% of the nebulized solution remains in the device after nebulization (as jet nebulizers require longer nebulization times, generally 30 minutes or more, the mentioned 15 minutes may be insufficient). In contrast, only 3.7% of the nebulized solution remains when using a mesh nebulizer (4). A survey based on 223 questionnaires revealed that the prevalence of vibration mesh nebulizers in China is less than 0.7% (5). Additionally, we need to focus on the accurate settings of ventilator-related parameters during antibiotic nebulization, particularly the positive end-expiratory pressure (PEEP) setting. The study mentions setting PEEP at 6 cmH_2_O, which may not meet the mechanical ventilation requirements for severe pneumonia patients with acute respiratory distress syndrome (ARDS). ARDS patients often require higher PEEP levels to reopen collapsed alveoli and facilitate gas exchange, typically ranging from 8 to 10 cmH_2_O, or even higher, and requiring dynamic adjustments (6, 7). If PEEP is rigidly set at 6 cmH_2_O using a “one-size-fits-all” approach, it may result in incomplete alveolar expansion, reducing the surface area of alveoli exposed to nebulized particles.

Finally, we still need to further focus on the duration of nebulized antibiotics and the selection of patient groups that benefit from them. (i) Under normal circumstances, the treatment course for ventilator-associated pneumonia (VAP) is 10–14 days, and for pneumonia caused by non-fermenting bacteria such as *Pseudomonas aeruginosa* and *Achromobacter baumannii*, short-course therapy is not recommended (8). Therefore, a 3-day nebulized antibiotic regimen may not fully meet the treatment requirements (1); (ii) when screening for patient groups that benefit from nebulized antibiotics, it may be more appropriate to define them as those with pulmonary infections caused by difficult-to-treat Gram-negative bacteria, as such patients have fewer options for intravenous antibiotics, and increasing the dosage may lead to liver and kidney dysfunction (9); (iii) the study grouping still needs further refinement. The use of intravenous antibiotics is the cornerstone of treating pneumonia because the lungs have a rich network of capillaries, and pathogens may spread throughout the body, which is why blood cultures are routinely taken from patients with severe pneumonia. Treating VAP with nebulized antibiotics alone does not comply with clinical standards, and its efficacy requires further validation (10).

## EDITOR'S NOTE

**Ed. Note**: The authors of the original article did not respond at the time of publication.
